# Use of Artificial Intelligence in Dental Implant Navigation Systems: A Scoping Review

**DOI:** 10.7759/cureus.100776

**Published:** 2026-01-04

**Authors:** Shankar S Menon, Shweta Ann Jacob, Alan Eldho Paul, Arun Kurumathur Vasudevan, Biju Balakrishnan, Maya Rajan Peter, Reshma Suresh

**Affiliations:** 1 Periodontics, Amrita School of Dentistry, Amrita Vishwa Vidyapeetham, Kochi, IND; 2 Dentistry, Amrita School of Dentistry, Amrita Vishwa Vidyapeetham, Kochi, IND

**Keywords:** artificial intelligence, computer-assisted surgery, dental implants, dynamic guidance, implant planning, machine learning, navigation systems, robotics

## Abstract

Artificial intelligence (AI) is transforming dental implant navigation systems, making implant surgeries more precise, efficient, and predictable. This scoping review examines how AI improves implantology, covering planning, surgery, and follow-up care, based on studies from 2019 to 2025. AI uses advanced computer programs to analyze 3D scans, automatically planning implant positions with high accuracy. During surgery, AI guides tools in real time, adjusting for jaw movements to place implants within a fraction of a millimeter of the plan. These systems reduce mistakes, shorten surgery time by up to 30%, and improve how implants bond with bone. AI also predicts long-term implant success by analyzing patient data, helping dentists plan better follow-up care. Despite these benefits, challenges remain. AI systems can be hard to understand, raising concerns about trust and reliability. They may work less well for some patient groups if trained on limited data, and strict regulations can slow their use in clinics. High costs also limit access, especially for smaller practices. Ethical issues, like protecting patient data and ensuring fair access to AI tools, are important to address. The review shows AI systems achieve very small errors, less than 2 degrees in angle and 0.5 mm in position, compared to traditional methods. Looking ahead, combining AI with technologies like augmented reality could make surgeries even more accurate, while predicting implant durability could improve outcomes. This scoping review highlights how AI can make implantology more personalized and tailored to each patient. Dentists are encouraged to use AI carefully, combining it with their expertise to provide better care. This work offers a clear guide for implantologists navigating the growing role of AI in dental implant surgery.

## Introduction and background

Dental implantology has progressed significantly, with advancements in technology improving the precision of implant placement [1]. Accurate implant positioning is critical, as errors can lead to complications like mechanical failures or gum issues [2]. Traditional freehand methods, relying on surgeon experience and two-dimensional (2D) X-rays, often produce inaccuracies, highlighting the need for better guidance systems [3]. In the 1990s, computer-assisted surgery introduced three-dimensional (3D)-printed guides to enhance accuracy, but these are rigid and can be disrupted during surgery [4]. This is compounded by the necessity of taking these 3D-printed guides, called Cone Beam Computed Tomography (CBCT) scans, multiple times if the treatment schedule is disrupted. By 2010, dynamic navigation systems emerged, using optical tracking to show drill positions in real time, though they still require manual calibration [5].

Dental implant placement traditionally relied on freehand techniques, which depend heavily on the surgeon’s experience and two-dimensional imaging, often resulting in significant inaccuracies such as angular deviations exceeding 5-10 degrees and linear errors greater than 2 mm, increasing risks of complications including nerve injury, sinus perforation, or implant failure [3]. This prompted the evolution toward computer-assisted implant surgery (CAIS), beginning with static surgical guides fabricated from preoperative CBCT scans; however, these rigid guides remain vulnerable to intraoperative disruptions, tissue shifts, or deviations during osteotomy, and may require multiple CBCT acquisitions if treatment plans change, adding radiation exposure and logistical challenges [4]. Dynamic navigation systems later emerged to provide real-time optical tracking, yet they still demand manual calibration and struggle with patient movement or soft-tissue interference [5]. Artificial intelligence (AI) now addresses these limitations by enabling adaptive, data-driven planning and intraoperative guidance that dynamically adjusts to anatomical variations and surgical conditions, markedly improving precision and predictability [5].

AI is now revolutionizing implant navigation by using advanced algorithms to analyze data and guide procedures [6]. AI, including machine learning techniques, processes 3D scans, digital impressions, and tissue data to plan optimal implant positions [7]. It automates tasks like outlining bone shapes before surgery, adjusts drill paths during procedures, and predicts long-term outcomes [8]. For example, AI can map bone structures with 96.4% accuracy, reducing planning time and errors compared to manual methods [2]. This allows both experienced and novice surgeons to achieve consistent results [9].

The demand for implants is growing due to widespread tooth loss, making efficient and accurate systems essential [10]. Conventional navigation is difficult in complex cases, such as thin jawbones or areas near sinuses, where plans may not match surgical conditions [11]. AI addresses this by predicting implant paths with high precision, minimizing risks like bone damage [12]. It also streamlines workflows, allowing clinics to treat more patients efficiently [13]. However, AI faces challenges, including unclear decision-making processes that raise ethical and legal concerns [14]. Regulatory approvals for AI systems, like robotic platforms, are often slow, especially in countries like India, owing to bureaucratic logjam, delaying their use [15]. Additionally, AI adoption is more common in wealthier regions of the world, potentially increasing global disparities in dental care access [16, 17].

This scoping review examines AI’s role in implant navigation, drawing on studies from 2019 to 2025 to highlight its benefits and challenges. AI’s dental applications began with diagnostic tools, such as detecting tooth decay, but expanded to surgical uses due to improved computing power [18]. Early AI studies showed high accuracy in planning implants using 3D scans, reducing reliance on subjective judgments [19]. Navigation systems have evolved from static guides to AI-enhanced tools that improve surgical precision [20]. For instance, robotic systems combining AI with mechanics achieve highly accurate implant placement [14]. This review uses recent studies to explore how AI improves planning, surgery, and follow-up, guiding dentists toward informed adoption. AI does not replace clinical expertise but enhances it, allowing dentists to focus on patient care while achieving precise, reliable results.

## Review

Methodology

This scoping review evaluates the role of AI in dental implant navigation systems. It aims to synthesize evidence on AI applications in implant planning, surgery, and follow-up, focusing on studies from January 2019 to September 2025 to capture recent advancements.

A comprehensive literature search was conducted across PubMed/MEDLINE, Scopus, Web of Science, and Embase, supplemented by Google Scholar and clinical trial registries (ClinicalTrials.gov, EU Clinical Trials Register) to identify relevant studies and ongoing trials. Search terms used include: ("artificial intelligence" OR "machine learning" OR "deep learning" OR "neural networks") AND ("dental implant*" OR "endosseous implant*") AND ("navigation system*" OR "dynamic guidance" OR "computer-assisted surgery" OR "robotic assistance"). Searches were limited to English-language publications from 2019 to 2025, aligning with the rise of AI in implantology. The last search was performed on September 30, 2025.

Studies were included if they: (i) were experimental or quasi-experimental, evaluating AI’s impact on navigation accuracy (e.g., angular/linear errors), efficiency (e.g., procedure time), or outcomes (e.g., osseointegration, complications); (ii) used AI methods like convolutional or recurrent neural networks; (iii) involved human, cadaveric, or simulated models, with computational validations as secondary sources. Exclusions included studies on AI diagnostics without navigation focus, non-implant dental topics (e.g., orthodontics), or non-empirical articles (e.g., editorials). Reviews were included to contextualize findings, but not for primary data.

Data were extracted on study design, AI methods, navigation outcomes, and barriers to adoption, using a standardized form to ensure consistency. Key themes included AI accuracy, clinical efficiency, and adoption challenges.

Discussion

The integration of AI into dental implant navigation systems has significantly advanced implantology, enhancing precision, efficiency, and patient outcomes [1]. AI plays a pivotal role in preoperative planning, intraoperative guidance, and postoperative evaluation. AI’s ability to process extensive datasets and automate tasks transforms implant surgery, but technical, ethical, and practical barriers must be addressed to fully harness its potential [2].

AI in Preoperative Planning

AI revolutionizes preoperative planning by automating the analysis of CBCT scans, a cornerstone of implant placement [2]. Traditionally, clinicians manually outline bone structures to determine implant positions, a time-consuming process prone to inconsistencies [3]. AI, particularly convolutional neural networks (CNNs), can analyze thousands of CBCT images to map alveolar ridges with high precision. For instance, a study achieved 96.4% accuracy in bone segmentation, surpassing human experts’ 85% concordance [2]. This automation reduces planning time from 45 minutes to approximately 8 minutes and detects subtle bone defects, such as buccal plate deficiencies, with 94% specificity, preventing complications like perforations [2].

AI also customizes implant plans by integrating data on bone density, jaw anatomy, and adjacent structures like nerves or sinuses [6]. This personalization is critical for complex cases, such as atrophic mandibles or sinus-proximate maxillae, where conventional planning often falters [15]. Studies show AI achieves 95% alignment with optimal implant trajectories, ensuring safer and more predictable surgeries [6]. By streamlining these processes, AI allows clinicians to focus on patient care rather than technical tasks, improving efficiency and reducing errors [10]. For example, AI-driven planning has been shown to expedite workflows, cutting chair time by 20-40 minutes, which supports higher patient flow [15].

AI in Prosthetically Driven Planning and Restoration

A critical aspect of successful dental implantology is the prosthetically driven approach, where surgical placement is guided by restorability to ensure optimal functional and aesthetic outcomes. AI significantly enhances this integration by incorporating prosthetic considerations into preoperative planning, such as simulating occlusal forces, biomechanical loading, and ideal prosthetic contours from intraoral scans and virtual wax-ups [10, 11]. For instance, AI algorithms can automate the design of crown morphologies, emergence profiles, and abutments that align with patient-specific occlusion and soft-tissue architecture, reducing biomechanical complications and simplifying the restorative phase. In full-arch cases, AI optimizes prosthetic fit by predicting stress distribution and peri-implant health, enabling more predictable long-term results [10]. This restoratively driven capability ensures that implant positions prioritize prosthetic success, allowing clinicians to achieve balanced occlusion and aesthetics while minimizing revisions.

AI in Intraoperative Navigation

AI enhances intraoperative navigation through dynamic systems and robotic assistance, both improving surgical accuracy [8]. Dynamic navigation systems use optical tracking to display drill positions in real time, but without AI, they struggle with tissue shifts or patient movement [21, 22]. AI-powered systems employ machine learning to adjust drill paths instantly, achieving angular errors as low as 1.2° compared to 3.8° in traditional dynamic navigation [4]. A study comparing AI-enhanced navigation to standard methods found tip errors reduced to 0.4 mm, even in challenging scenarios involving metallic restorations that distort imaging [13].

Robotic systems integrate AI with mechanical precision to guide implant placement [14]. These systems, such as the Yomi platform, which is a Food and Drug Administration (FDA)-cleared robotic system developed by Neocis for dental implant surgery, use algorithms to maintain drills within 0.1 mm of planned paths [14]. A meta-analysis of 14 in vitro studies (n=1,056 implants) reported robotic systems achieving an average accuracy of 0.61 mm, compared to 1.07 mm for static guides, with superior performance in posterior regions [3]. In a cadaveric study of 120 zygomatic implants, AI-guided robotics preserved 99% of sinus membranes, minimizing the need for bone grafts [4]. However, robotic systems can be fatiguing for surgeons if interfaces are not user-friendly, highlighting the need for ergonomic improvements [5].

AI also empowers less experienced clinicians, enabling them to achieve expert-level precision [16]. A clinical audit of 245 patients showed AI-assisted navigation ensured 98% adherence to preoperative plans, even for novice surgeons [17]. This capability could expand access to high-quality implant surgery in underserved regions, addressing global disparities in care [15]. For instance, AI’s ability to compensate for surgeon inexperience supports its potential to democratize expertise, making advanced implantology more accessible [16].

AI in Postoperative Evaluation

AI’s predictive capabilities extend to postoperative care, forecasting long-term implant success by analyzing imaging, genetic, and biomechanical data [6]. One study used AI to predict 5-year implant survival with 92% accuracy, outperforming traditional bone density assessments [7]. Another analysis of 312 implants identified stress patterns linked to marginal bone loss with 88% sensitivity, enabling tailored follow-up plans [8]. This proactive approach reduced corrective surgeries by 22% in high-risk cases [9]. In full-arch restorations, AI designs prosthetic contours to optimize fit and peri-implant health, enhancing long-term outcomes [10]. These tools make postoperative care more efficient, identifying potential issues early and improving patient outcomes [11].

AI’s ability to integrate diverse data provides a comprehensive view of implant performance, supporting better clinical decisions [12]. For example, AI-driven analytics can stratify patients by risk, allowing clinicians to prioritize intensive monitoring for those with higher chances of complications [13]. This targeted approach enhances patient satisfaction and reduces the burden of unexpected revisions, making postoperative management more efficient [14].

Challenges in AI Adoption

Despite the substantial advancements introduced by AI in dental implant navigation, several challenges continue to hinder its seamless integration into everyday clinical practice. One of the most prominent concerns is the “black box” nature of many AI models, particularly deep learning systems, which generate outputs without providing transparent reasoning [1]. This lack of interpretability limits clinician confidence, especially when implant placement involves anatomically critical areas such as the inferior alveolar nerve or the maxillary sinus [1]. Although explainable AI tools such as SHapley Additive exPlanations (SHAP) can help identify variables that influence algorithmic decisions, they do not fully resolve medico-legal concerns about accountability in cases where AI-assisted decisions may contribute to surgical errors. This ambiguity raises fundamental questions about whether responsibility lies with the clinician, the system developer, or the institution deploying the technology [6].

Another significant challenge is the issue of dataset bias, which limits the generalizability of current AI systems [7]. Many AI-powered navigation tools are trained on datasets that lack diversity, often focusing on ideal bone conditions, specific imaging protocols, or populations from limited geographic regions. When such systems are applied to patients with different craniofacial anatomies, systemic risk factors, or severe ridge resorption, their accuracy can decline markedly [7]. This problem is amplified in developing countries, where imaging equipment, exposure parameters, and anatomical characteristics differ from those represented in training datasets. As a result, the performance of AI systems may inadvertently contribute to unequal standards of care, contradicting the aim of using AI to democratize high-precision implantology [7].

Regulatory hurdles further complicate AI adoption. Healthcare regulators such as the FDA require extensive clinical validation and continuous performance monitoring before approving AI systems for medical use [14]. This process becomes even more complex when dealing with adaptive learning models that update their behavior over time, making it difficult for regulatory agencies to certify a system whose outputs may evolve beyond its originally approved version. Variations in regulatory efficiency across regions contribute to delays in AI availability, with some technologies widely used in Europe still awaiting approval in India or other markets. Such inconsistencies create geographical imbalances in the accessibility of AI-driven implant navigation tools [15].

Financial constraints represent another substantial barrier. AI-driven navigation and robotic systems involve high acquisition costs, often ranging from USD 150,000 to 250,000, along with ongoing expenses for maintenance, software updates, staff training, and infrastructure upgrades [15]. These costs can be prohibitive for smaller clinics or practices in low-resource settings. Even when clinics are able to invest in such technologies, the return on investment depends heavily on patient volume, the complexity of cases treated, and reimbursement policies. Without government support, insurance coverage, or shared-use models, AI technologies may remain concentrated in large, affluent institutions rather than becoming universally accessible tools [15].

Robotic systems like Yomi (Neocis) involve substantial upfront costs, plus ongoing expenses such as per-implant fees, software updates, maintenance, and staff training [15]. These costs can be prohibitive for smaller practices, potentially limiting adoption to high-volume or affluent clinics. While robotic assistance achieves superior placement accuracy, the marginal improvement in long-term implant survival rates remains limited in short-term studies, with overall failure rates low across techniques. Revisions, when needed, can cost patients USD 3,000-5,000 per implant (including removal, grafting, and replacement), but the absolute risk reduction may not offset upfront expenses for low-volume providers. However, potential savings arise from increased case volume, reduced chair time, fewer complications, and enhanced practice differentiation, attracting more patients [15].

Ethical and legal considerations also play a significant role in slowing AI integration [16]. AI systems depend on large datasets, often stored or processed through cloud-based platforms, raising concerns about data privacy, security, and patient consent. Many patients may not be aware that their imaging data could be used to train commercial algorithms, potentially violating privacy regulations. Cross-border data transfer complicates compliance further, especially when AI vendors store or analyze data in jurisdictions with different privacy standards. Cybersecurity threats, such as hacking or data breaches, introduce additional risks that clinics must manage diligently [16].

Another concern relates to the potential over-reliance on AI, which may contribute to a gradual erosion of clinicians’ manual skills [17]. While AI enables novice clinicians to achieve expert-level precision, heavy dependence could limit their ability to adapt when unexpected intraoperative complications arise or when technology fails. Although studies show that clinicians override AI recommendations in approximately 7% of cases, demonstrating the preservation of clinical judgment, there is still concern that over-automation may diminish intuitive surgical decision-making over time, particularly among younger practitioners trained primarily on AI-assisted workflows [17].

A further limitation is the lack of comprehensive long-term clinical evidence confirming the superiority of AI-enhanced implant placement. While numerous in vitro studies and short-term clinical trials demonstrate improved accuracy and reduced deviation, evidence connecting AI navigation to improved long-term outcomes - such as enhanced osseointegration, reduced peri-implant disease, or lower failure rates - remains limited [6]. Without robust, multicenter randomized controlled trials, many clinicians remain understandably cautious about fully integrating AI into their surgical protocols. The absence of standardized guidelines from professional bodies further delays widespread clinical adoption [7].

Future directions

The future of AI in dental implant navigation is poised to reshape the landscape of implantology, offering innovative applications that extend well beyond the capabilities of current systems. One of the most promising avenues is the integration of AI with augmented reality (AR) and mixed reality (MR) technologies [8-10]. These systems have the potential to project real-time implant trajectories, anatomical landmarks, and virtual drilling paths directly onto the surgical field, reducing dependence on physical tracking devices and enabling surgeons to maintain continuous visual focus on the operative site [10]. Early pilot studies have demonstrated the feasibility of AR-assisted implant placement with accuracy levels approaching 0.3 mm, suggesting that future systems may combine AI-driven planning with immersive 3D visualization to create hybrid navigation environments that enhance precision while simplifying workflow [10].

Another major area of development involves the evolution of robotic implant surgery [11]. While current robotic systems provide assistance by guiding drill angulations and depths, future generations are expected to become increasingly autonomous. AI-driven robotics could eventually perform entire sequences of implant placement with minimal operator intervention, coordinating multiple drills, adjusting for patient movement, and modifying plans intraoperatively based on real-time feedback from sensors and imaging systems. For full-arch reconstructions, such systems may be capable of placing multiple implants simultaneously, potentially reducing surgical time from several hours to under 90 minutes [11].

Advances in machine learning algorithms are also expected to transform how clinicians plan and predict implant outcomes [12]. Future models will likely incorporate multimodal datasets, including CBCT images, intraoral scans, clinical photographs, patient genetics, bone quality metrics, occlusal forces, and even microbiome profiles, to create personalized risk maps for each patient [12]. With such comprehensive datasets, AI may be able to forecast long-term implant behavior, predict peri-implant bone loss trajectories, identify patients at elevated risk of complications, and recommend tailored follow-up schedules [10, 12].

Another emerging direction is the development of federated learning systems, which allow AI models to learn from data distributed across multiple clinics and countries without compromising patient privacy [12]. This approach could overcome current limitations of dataset bias, helping to create globally representative models that perform consistently across diverse populations, imaging systems, and anatomical variations. Such collaborative models could significantly improve the reliability and fairness of AI-driven navigation, especially in regions with limited access to high-quality training datasets [12, 13]. Interoperability is also expected to improve substantially in the coming years. Currently, one of the major bottlenecks in AI adoption is the lack of seamless integration between CBCT machines, intraoral scanners, surgical navigation devices, and practice management software. Future platforms may adopt standardized protocols and open-source frameworks that allow effortless data exchange between devices, enabling uninterrupted digital workflows from diagnosis to final prosthesis delivery [13].

AI-driven simulation and training tools represent another transformative direction [14]. Future implant education may involve virtual reality (VR) platforms where trainees perform AI-assisted mock surgeries, receiving real-time feedback on angulation, depth control, and hand stability. These simulators could democratize access to advanced implant training, particularly for clinicians in regions without robotic systems or high-end navigation units [14].

Finally, future research must focus on large-scale, multicenter clinical trials that evaluate not only surgical accuracy but also long-term patient outcomes [15]. These studies should include diverse populations, complex anatomical conditions, and real-world variables such as clinician experience and system ergonomics. As stronger evidence accumulates, AI may evolve from an optional adjunct into a standard-of-care tool, integrated seamlessly into every stage of implant diagnosis, planning, placement, and maintenance. With continued interdisciplinary collaboration among clinicians, engineers, policymakers, and industry partners, AI has the potential to revolutionize implant dentistry, making treatment safer, faster, more predictable, and universally accessible [15].

Implications

AI transforms dental implant navigation by converting complex data into precise actions, reducing errors and enhancing outcomes. From automating planning to guiding surgeries and predicting results, AI makes implantology more reliable, particularly in challenging anatomies like zygomatic arches or resorbed ridges [20]. However, addressing transparency, bias, cost, and ethical concerns is critical to ensure equitable benefits. Clinicians should view AI as a partner, leveraging its capabilities while preserving their judgment to deliver patient-centered care [22]. As AI evolves, it promises to make dental implants more predictable and accessible, reshaping the field with scientific precision [23]. By balancing innovation with rigorous validation, AI can elevate implantology to new standards of excellence, ensuring safer and more effective treatments for patients worldwide. Ultimately, AI’s integration into navigation systems heralds a future where dental restoration is both highly accurate and widely attainable [24].

## Conclusions

The incorporation of AI into dental implant navigation systems marks a significant advancement in implantology, offering improved accuracy and better patient outcomes. This scoping review highlights AI’s ability to enhance every stage of implant surgery, from planning to execution to follow-up care. By automating complex tasks and providing precise guidance, AI makes implant placement more reliable and efficient. Combining AI with technologies like AR could allow surgeons to see implant plans directly on the patient’s jaw, improving accuracy further. Future systems might place multiple implants at once, cutting surgery time significantly. However, these advancements need thorough testing through large clinical studies to ensure they work reliably in real-world settings. Collaboration between dentists, engineers, and policymakers is essential to create guidelines that make AI safe, affordable, and fair for all patients. With continued research and development, AI promises to make dental implants more accurate, efficient, and accessible, transforming the field and ensuring better smiles for patients worldwide.
